# Assessment of the status of improved F&E trachoma control practices among children of agro-pastoralists in Southern Ethiopia: a mixed design survey using theory of triadic influences

**DOI:** 10.1186/s12889-023-15438-9

**Published:** 2023-03-23

**Authors:** Serawit Lakew, Genet Asefa, Zerihun Zerdo

**Affiliations:** College of Medicine and Health Sciences, Arba Minch University, Arba Minch, Southwest Ethiopia Ethiopia

**Keywords:** F&E, Trachoma, Children 1–9 years, Rural community, South Ethiopia

## Abstract

**Background:**

Ethiopia is one of the countries with heavy trachoma burdens states globally. More than 75 million people in Ethiopia live in the trachoma endemic zones. Most populations with neglected tropical diseases (NTDs) live in hard-to-reach residences because of landscape and socio-cultural variances. This survey assessed the status of improved Face hygiene and Environmental cleanliness (F&E) trachoma control practices in children 1–9 years of age.

**Methods:**

A mixed-method study design was applied concurrently. Enumeration was done through interviews using the standard tool and observational technique. Focus Group discussions (FGDs) and Key informant Interviews (KIIs) were used to conduct the qualitative arm. Confounders were controlled by modeling with multivariable logistic regression.

**Results:**

For the Quantitative survey: The response rate was 99.8% of participants. The proportion with improved practice was 8%. About 13.9% of a child washed their faces and were visibly clean. About 15.2% of the households had an observable clean environment. High Wealth index, Perceived ability, knowledge about trachoma transmission from person to person, and stance toward preventive behavior were associated with improved practices. The odds of having improved F&E practice were 67% lower for those who reported positive normative preventive behavior than negatives. Qualitative arm: Some key informants reported village dwellers’ shortage of basic knowledge; attitude and behavioral change for improved hygienic practices are the challenges. Inhabitants, including elder children, are aware of the hygiene issue though they do not practice it or have no intention to practice it.

**Conclusions:**

Improved F&E practices were much lower in the study region than the regional plan to achieve.

## Background

Globally, around 200 million people were estimated to exist in trachoma-endemic areas and are at risk of developing loss of sight [1]. Transmission of the infectious agent and ocular strains of Chlamydia trachomatis takes place from person to person via flies or hand contamination, clothing, and bedding [2, 3].

Currently, it has been reported that trachoma is responsible for about 3% of the world’s blindness [4]. Globally, an estimated 2.2 million people are reported to be visually impaired as a result of trachoma, of which 1.2 million lost their sight [4]. About 21 million had active trachoma and 7.3 million require surgical intervention for trachomatous trichiasis [4, 5]. Blinding causes of trachoma are common in many of the disadvantaged and remotest areas of 51 countries in Africa, Asia, Central, and South America, Australia, and the Middle East [4]. Africa is the worst affected continent, where 18 million people with active trachoma and about 3.2 million cases of trichiasis are believed to live [4, 5].

Ethiopia and South Sudan together encompass the highest prevalence of active trachoma. In some areas of the two countries, active trachoma exists in more than half of children aged 1–9 years and trichiasis affects more than 10% of adults aged 15 + years [5]. Trachoma is endemic in Ethiopia, with the highest prevalence of both active and potentially blinding trachoma [6]. It is the 2^nd^ known cause of loss of sight following a cataract [7].

The result of the meta-analysis shows Ethiopia’s national prevalence of active trachoma in children aged below 15 years was 26.9% with a high prevalence in the current study regional state (35.8%) [8]. Among children aged 1–9 years, the prevalence of TF ranges from 12 to 45% [9]. Substantial regional disparities were also seen in the existence of active trachoma from one district to another in that the prevalence in 1–9 years children ranges from 1.95% in the Mihur Aklil district to 25.4% in Damot Pulasa of Wolaita, though it has shown a declining trend over time in SNNP and Oromiya regional state as reported by both AMREF and WASH project [10, 11].

According to Water, Sanitation and Hygiene (WASH) recent Survey report, Only 20% of the households in the 52 districts of the former Southern Ethiopia region included in the study had access to clean water within a 30 min journey and 3% had improved latrine coverage [10] and a systematic review report observed a significant association between high prevalence and unimproved practices in Ethiopia [8]. Moreover, TF was more common in early childhood (1-3 years) than in older [10].

An extensive task has been done in implementing and tracking S and A components of the SAFE strategy in endemic countries [12]. Mass Drug Administration (MDA) of antibiotic therapy has demonstrated effectiveness in dropping the prevalence of active trachoma and ocular C. trachomatis infections [12]. This intervention has not yet yielded the elimination of diseases as a public health problem in areas where the SAFE strategy has been implemented [13]. Additional expansion and integration of non-chemotherapeutic preventive interventions are required to strengthen trachoma control practices [13]. The implementation of the F and E components of the SAFE strategy was reported inconsistently [14].

Though the F and E components are critical for interventions designed to address key mechanisms of behavior change, the involved stakeholders will be challenged to address their goal in the absence of collaborative effort that includes leadership commitment and community involvement [15]. Inter-sectorial collaboration is needed for a better outcome to support those already working in water and hygiene, sanitation, education, and health promotion [15]. Hence, this survey could show the coalition working on Trachoma elimination in Ethiopia to evaluate where their implementation reached in the very local and agro-pastoral regions of Southern Ethiopia. The result of this study can also be used by the stakeholders involved in the implementation of the SAFE strategy for trachoma elimination in the region to evaluate the F and E portion of the intervention.

## Methods

### Study design and setting

A mixed-method design was used for the survey. Data was collected simultaneously. The study district is located some 530 km distance from Addis Ababa, the capital city of Ethiopia. The district is divided into 14 “kebele”, the smallest administrative unit in the country. The total estimated population of the district was 94,350 [16]. The study was undertaken between January 15 and Feb 4/2021.

### The theoretical and conceptual frameworks

**Fig. 1:**
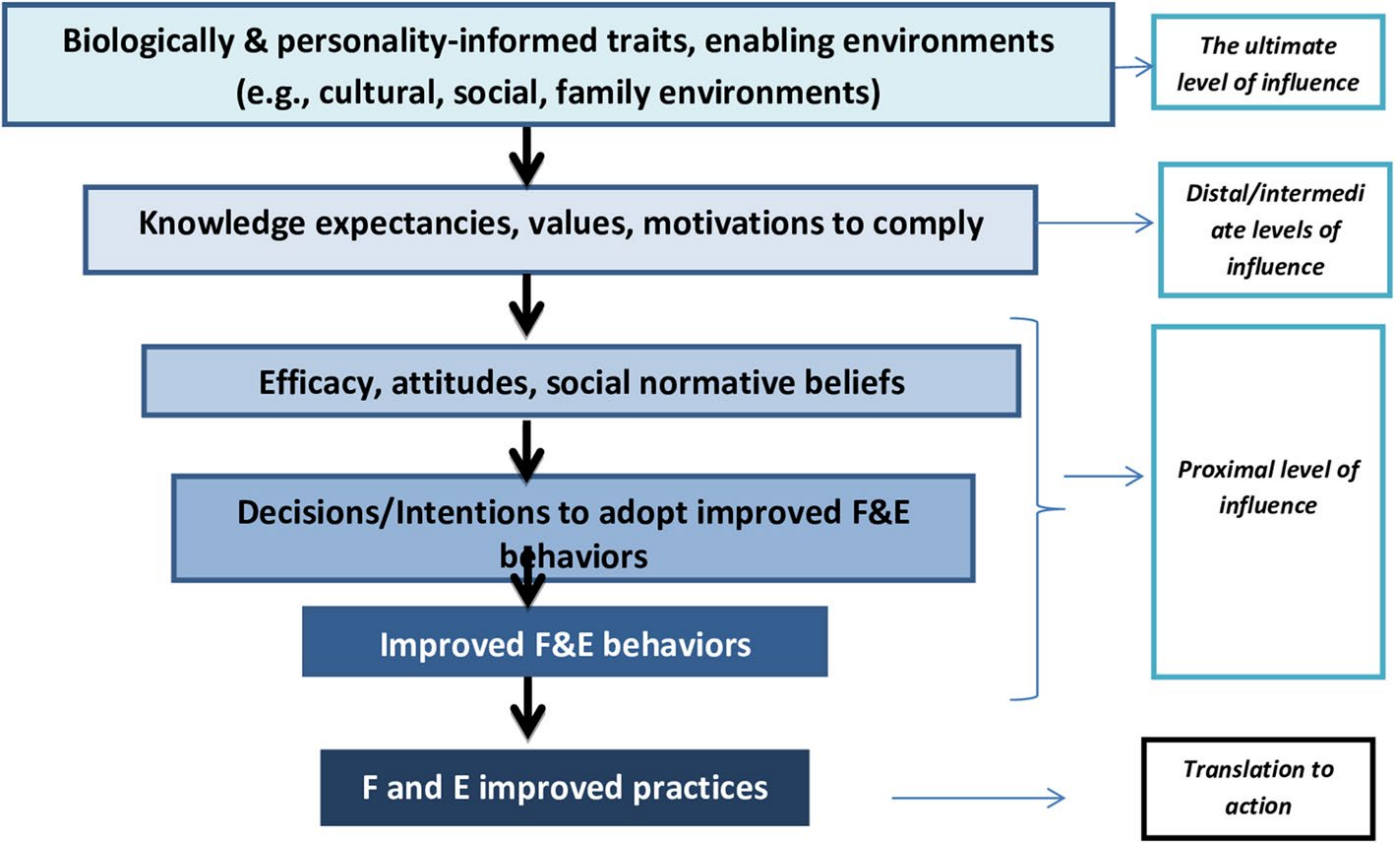
Adaptation of the Theory of Triadic Influence (TTI) showing the relationship between improved F and E practices for trachoma control and antecedent factors in the model

Among many behavioral change factors used to intervene in the assessment techniques, some of the factors are more achievable in bringing change over others. These behavioral change factors were intrinsic to the characteristics of the individual factors to influence on behavior. Some techniques operate at a more distal level to impact on bringing change when intervening by isolation. Others impact at a more proximal level of stimulus and are more likely to influence. Based on the Theory of Triadic Influences, several tiers of inspiration exist and account for factors that have a straight and indirect impact on behavior and actual practices [17]. The lowest and most influential tiers (such as the proximal level of stimulus) are efficacy (such as self and collective), stance, and social normative beliefs. They directly impact the decisions/intentions either to act or not to act in a certain way. More remote factors are values, knowledge of a person, and motivations to do that influence only moderately behavioral alterations. The last was the decisive level of influence that include factors related to biologically and personality-informed traits, and cultural, social, and/or family environments (such as enabling settings) [17–19]. So, all these factors influence the behavior change practices of the inhabitants (Fig. 1).

### Sample size and sampling procedure

#### The quantitative arm

**Fig. 2:**
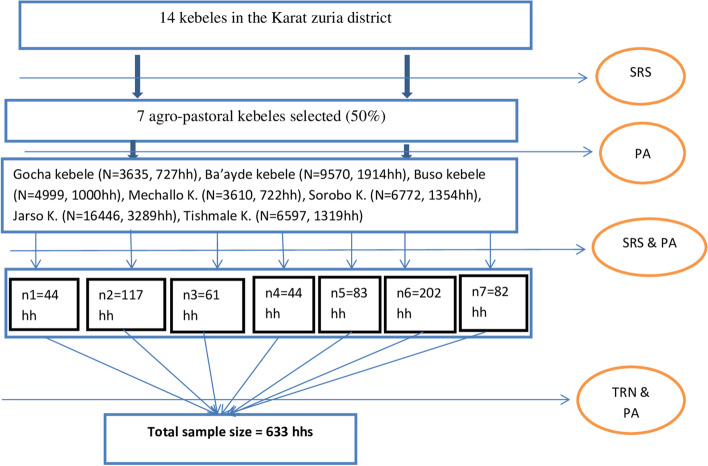
Sampling procedure and selection of representative participants in the study region. Note: SRS/TRN: simple random sampling/Table of Random numbers, PA: proportional allocation of the participant, HH: total household number, N: total population, n: number of households selected in each kebele, K: kebele administration

Out of the 14 agro-pastoralist kebeles under Karat Zuria District, seven were randomly selected and included in the study. Households from each selected kebeles were selected randomly using a sampling frame which was obtained by household listing before data collection. Sample size allocation to each kebeles was done using a proportional allocation technique based on listed households. The household head was a participant interviewed from a household. To get an overall adequate sample size for this study, the formula used was single population proportion formula computed using online openepi.com by considering the proportion of face washing in central Ethiopia (*p* = 52%) from the previous study [20], design effect of 1.5 and 10% non-response rate, 95% confidence level, and 5% precision. accordingly, the total estimated sample size was 633 households (Fig. 2).

#### For qualitative sampling

Two FGDs and three KIIs were carried out in the district. FGD discussants were village dwellers of both sex and mature age (30–70 years). The number of people involved in the FGDs ranges between six and seven. KII participants were selected from ORBIS international Ethiopia country office, the Communicable Diseases Prevention and Control (CDC) focal person from the district health office, and the zonal Neglected Tropical Diseases (NTDs) focal person. The KIIs were selected based on their experiences working in water and sanitation in the area.

### Measurement

Enumeration was done using pretested and structured data collection tool which contains biologically and personality-informed traits, enabling environments, knowledge expectancies, values to practice, motivations to comply, efficacy, attitudes, social normative beliefs, decisions or intention to practice better F&E hygienic behaviors, and improved F&E behavior, adapted from health behavioral model for both quantitative and qualitative methods [18, 19]. Improved F&E practices in this context was operationalized as a composite variable where all ‘yes’ response to four items, such as a history of face washing today to all the children at home during data collection, visibly clean face to all the children at home while data collection, history of disposing of human feces properly, and no any visible feces in the compound or clean compound as observed by data collectors. The questionnaire was developed in English, Amharic, and Konsigna (local language) versions to be used in interview data collection.

### Statistical analysis

**Quantitative arm:** Data was collected electronically using the Open Data Kit (ODK) by mobile tablet. Seven Bachelor of Nursing fresh graduates were hired as data collectors, received 5 days of training, and collected data in 1-month duration. Data was received into the STATA 14 statistical software package from the server for processing and analysis. Tables and graphs were used to present the descriptive result. To compute the wealth index, a principal component analysis was done. A binary logistic regression model was fitted to see statistical associations between improved F&E trachoma control practices and its predictors. The steps were used to compute this in that the first binary regression model was fitted to each independent variable where a *P* value < 0.25 was considered for the existence of associations. Candidate variables (*P* < 0.25 in binary) under the domains of independent factors were fitted into the multivariable logistic regression model to make the final adjustment. *P* value < 0.05 was considered significant in the adjusted associations. Model fitness was done using Hosmer and Lemeshow goodness fittest [21]. Variable collinearity was checked. **Qualitative arm:** Data recorded by tape-recording upon collection from FGDs and KIIs. Record translated and transcribed word by word, coded, categorized, and analyzed thematically. All the steps were done through a manual process. Both arms were reported side to side in the result report.

### Data quality control

To maintain data quality different efforts were done starting from properly designing and pre-testing the questionnaire and intensive training of data collectors and supervisors. Separate training was given for expert qualitative data collectors on FGD and KIIs using the pre-prepared interview guide. The data collection tool used in the quantitative and qualitative arm was the standard tool that was adjusted to the local context [19]. The collected data were edited by data collectors and supervisors every night before submission to the server. Three data collection language options were used, including the local language (Konsigna) for both arms.

## Results

### Basic characteristics

Six hundred thirty-two (632) households were involved in this study with a 99.8% of response rate. The mean age of the household head (± SD) was 31.5 ± 9.6 years. Nearly more than four-fifths of the household head were married (85.1%) and completed primary school (82.3%). About ninety-three percent (92.7%) of the study participants were followers of the Protestant Religion. The major occupation of the households was agro-pastoralist as agrarian and pastoral (71.5%) and in the crowded (≥ 5 family members) family (64.2%, mean ± SD, 6.83 ± 3.1) (Table 1).

**Table 1:** Basic characteristics of study participants (*n* = 632) for improved F&E trachoma control practices at Karat Zuria District, Konso Zone, Southern Ethiopia, from January to July 2021

**Variables**	**Number**	**Percent**
Age in years of participant		
18–24 years	130	20.6
25–34 years	276	43.7
35 +	226	35.8
Marital Status		
Married	538	85.1
Single	80	12.7
Others^a^	14	2.2
Religion of participant		
Christian Protestant	586	92.7
Others^b^	46	7.3
Family number		
Five or less	226	35.8
More than 5	406	64.2
Education of participant		
Primary school (1–8)	520	82.3
High-school (9–12)	94	14.9
More than high-school (> 12)	18	2.8
Wealth Quintile of the household		
Lowest	104	16.5
Low	126	19.9
Middle	150	23.7
High	182	28.8
Highest	70	11.1
The major occupation of the participant		
Farmer	452	71.5
Merchant	62	9.8
Government employed	42	6.6
Others^c^	76	12

Others^a^ = widowed, divorced marital status

Others^b^ = orthodox, Muslim, catholic religions

Others^c^ = daily laborer, housewife, student, NGOs employed

### Water sources and toilet facilities of the household

#### Quantitative arm

Surface water (rivers and streams) is reported as a major source of water for drinking, cooking, and washing for 92% of households. Nearly four-fifth (78.1%) of the households travel more than 30 min to fetch water. Most of the households use unprotected water sources (84.5%). About two-thirds of the household (34.8%) had no toilet facility yet so they were defecating in the field or bush (Table 2).

**Table 2:** Water sources and toilet facility of the households (*n* = 632) for improved F&E trachoma control practices at Karat Zuria District, Konso Zone, Southern Ethiopia

**Variables**	**Number**	**Percent**
Water sources of the household		
River	500	79.1
Stream	84	13.3
Others	48	7.6
Time to fetch water (to and from source of water)		
Below 30 min	138	21.8
30–60 min	222	35.1
More than 1 h	272	43.0
Water source protected or unprotected		
Protected	96	15.2
Unprotected	536	84.8
Toilet facility type		
Pit latrine with slab	248	39.2
No facility or bush or field	220	34.8
Pit latrine without slab/open pit	136	21.5
Ventilated improved pit latrine	28	4.4

Others = pool water and borehole water

#### Qualitative arm

The FGD discussants reported a shortage of clean water supply sources and latrines in the Woreda. The majority justified being the dry geographic area of the study locality is the main reason for shortages of water from underground. Financial constraint to constructing groundwater sources is another mentioned. Open defecation is widely practiced by adult members of the community and is one of the challenges in convincing the family to construct their latrine. Discussants reported as follows:

‘Except few areas in Karat zuria ‘woreda’, water shortage is a serious problem. Without community water services improvement, it is more difficult to improve the practices of F&E. The land is dry where deeply rooted water table is a challenge to do by the capability of inhabitants. Shortage of rivers is another to be seen in the district and zone as a whole. It was difficult to get water easily though some of the families tried to dig into the land very deep….’ (FGD discussant 8)

‘Some health facilities have water supply problems. For example, one health center (Soroba Health Center) and one health post in the district had no water supply among others. Others had supply but not adequate and continual yet. Imagine a health facility without a water supply. I do not consider doing most medical procedures at the health facility in the absence of water. Some kebele had tap water, some others are living without access to clean water. In my opinion, the problem of water access, in general, is a leadership commitment and economy though geography is somehow a factor. Water is available deep in the ground if an advanced machine digger could be used. Collaboration with funding organizations and leadership commitment should strongly be together to alleviate the community water supply problem…’ (FGD discussant 5)

‘Most households do not own latrines. The major problem of not using a latrine is due to the normalized habit of open defecation which is associated with past practices by the elders in the kebele, most commonly seen among ‘Jarso kebele’ dwellers. No one denies open defecation in the locality as bad habit…’ (discussant 4)

### Knowledge of trachoma and its transmission

**Fig. 3:**
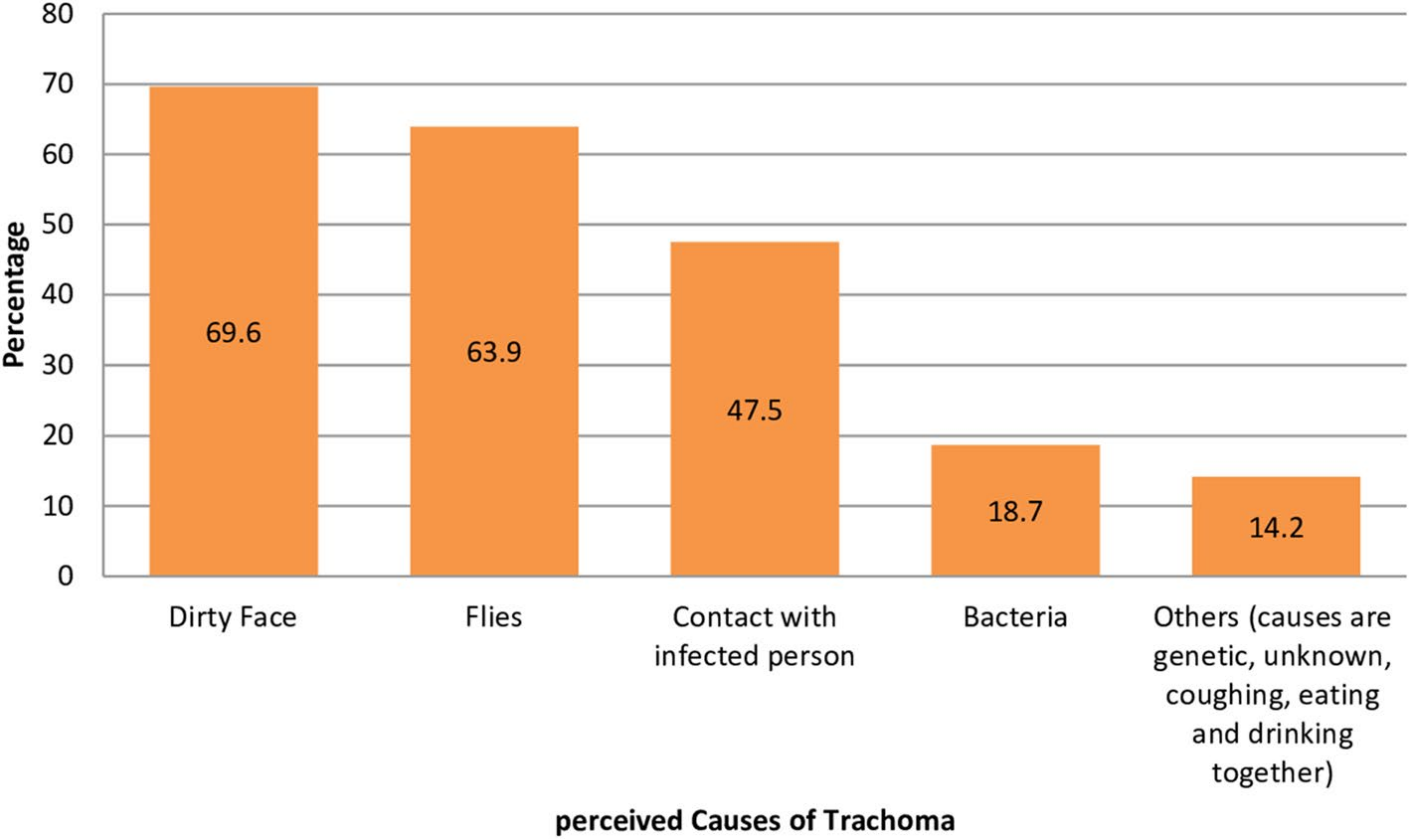
Perceived Causes of Trachoma by the study participant (*n* = 632) for improved F&E trachoma control practices in Southern Ethiopia

**Fig. 4:**
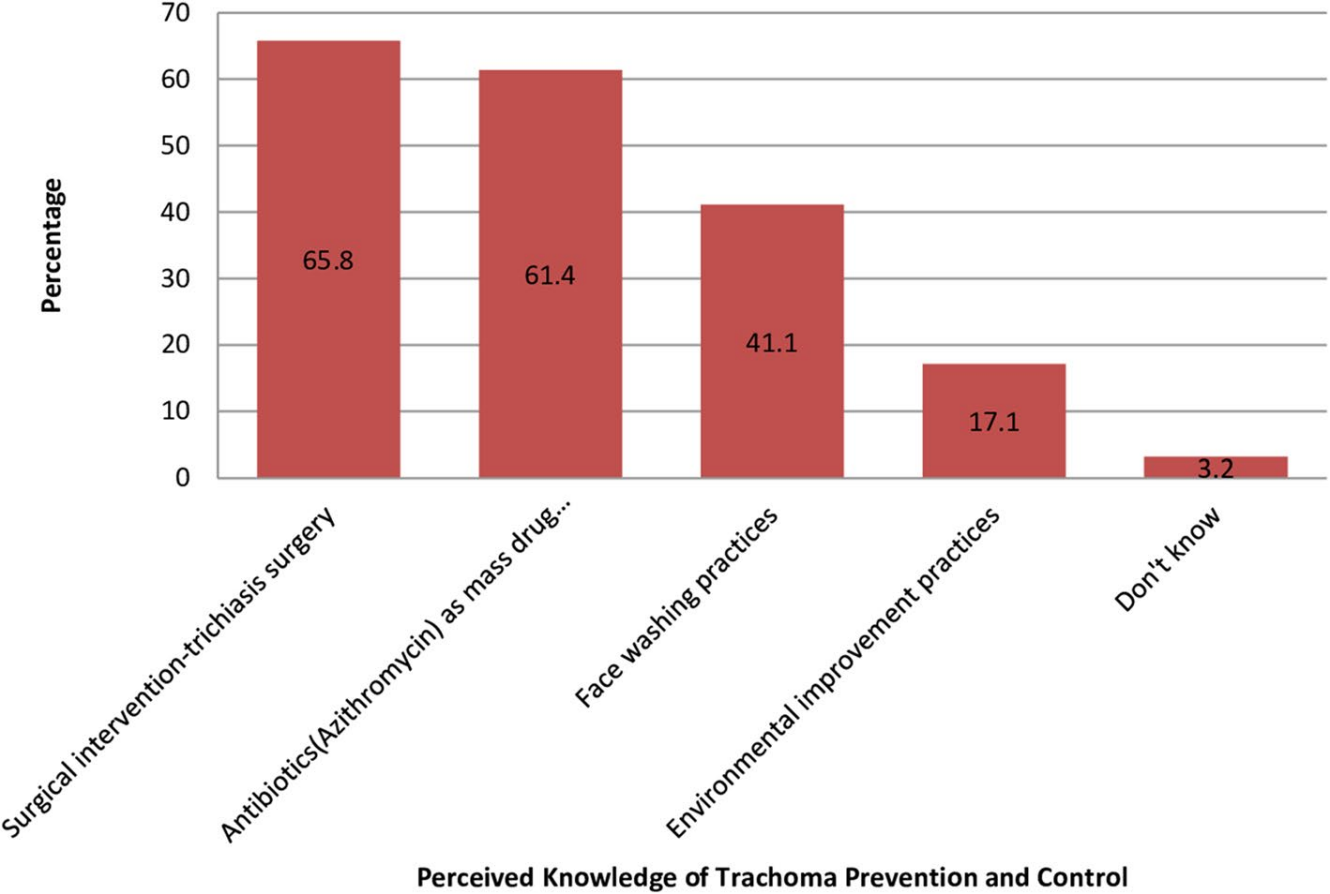
Perceived Knowledge of Trachoma prevention and control by the study participants (*n* = 632) for improved F&E trachoma control practices in Southern Ethiopia

About four-fifths of the study participants had awareness of trachoma. Among these, about seven out of ten believed that trachoma can cause blindness over time. The majority (70.6%) believed that trachoma can be transmitted by flies with feces to face contamination. But, only one out of ten (9.2%) believed that trachoma transmission occurs through flies with feces on faces, touching the eye with a contaminated finger, Sharing a washcloth, Shawls, towels, bed sheets, and pillows. Around 96.8% of the participant reported at least one manifestation of active trachoma, such as red eyes, eye rashes (corneal ulcer), watery eyes, or poor eyesight. Only a few (6%) reported all four common signs. Red eyes and eye rashes were reported signs by the majority (65.8 and 61.7%), respectively. Few participants believed that bacteria causes trachoma (18.7%). Seven out of ten believed trachoma is caused by a dirty face (69.6%) and six out of ten by flies (63.9%). Few inhabitant participants believed that trachoma can be prevented and controlled through improved environmental practices (17.1%). Slightly more than six out of ten believed that surgical interventions (65.8%) and mass drug administration to the children (61.4%) are keys to controlling trachoma in the locality (Table 3, Figs. 3 and 4).

**Table 3:** Context-specific risks and knowledge of trachoma and its transmission among study participants (*n* = 632) for improved F&E trachoma control practices at Southern Ethiopia

**Variables**	**Number**	**Percent**
Ever heard about trachoma		
Yes	500	79.1
No	132	20.9
Trachoma causes blindness		
Yes	440	69.6
No	192	30.4
Trachoma is transmitted from person to person by		
Flies	446	70.6
Eye rubbing by contaminated fingers	428	67.7
Sharing a towel or washcloth	288	45.6
Sharing Shawls, towels, bedsheets, and pillows	142	22.5
Others(other insects, unknown, water)	20	3.2
Signs of Trachoma on infected child		
Red eyes	416	65.8
Eye rashes/scarring eyelid	390	61.7
Watery/discharging nose	264	41.8
Poor vision/impairment/blindness	126	19.9
Don’t know	20	3.2

### Risk perception and attitude factors for improved F&E practices

#### Quantitative arm

**Fig. 5:**
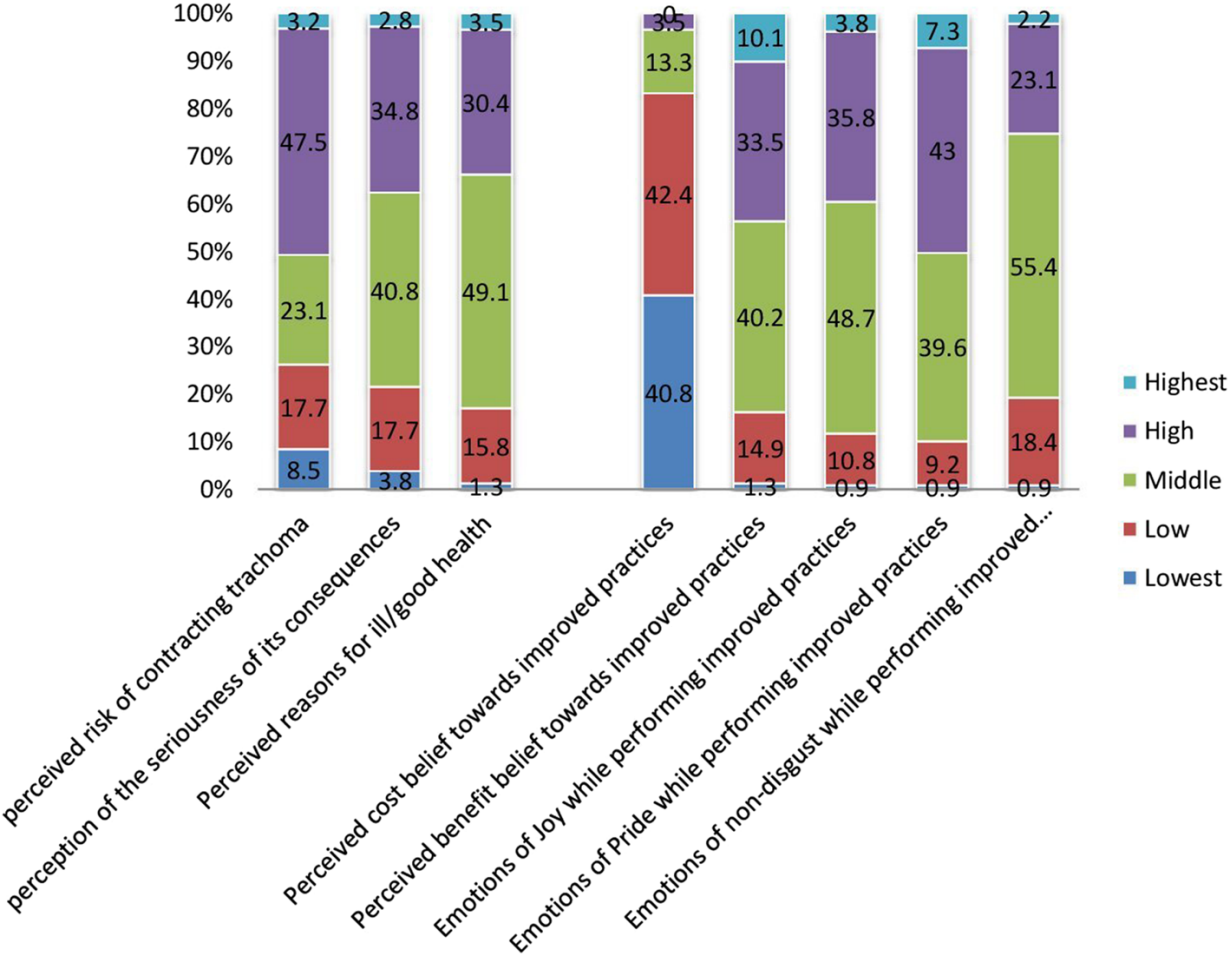
Risk perception and attitude factors of the study participants (*n* = 632) for improved F&E trachoma control practices in Southern Ethiopia

Among the respondents included in the study, almost half (50.6%) had either high or highest perceptions of contracting trachoma in the locality. Nearly a third of the participant feels the seriousness of the consequences (37.7%) of encountering trachoma and had perceived reasons for ill/good health (33.9%). A large number of participants reported as had a ‘medium/neutral stance’ to ‘perceived benefit belief towards improved F&E practices (40.2%) to prevent trachoma’, emotions of joy while performing improved F&E practices (48.7%)’, emotions of non-disgust while performing improved F&E practices (55.4%). Participants ‘perceived cost belief towards improved F&E practices (83.2%)’ was reported low (Fig. 5).

#### Qualitative arm

FGD also tried to see attitudinal problems for improved practices among the inhabitants, including children. Many discussants reported elders unimproved practices influenced children to continue with bad hygienic practices. One informant reported as follows:

‘In my opinion, attitude is another problem in the family. Face-washing skill is not a problem. Every family can wash their child’s face and train the child how to properly use the latrine. The problem is a low attitude towards hygienic practices by parents and elderly children. I suggest that for better improvement of hygienic practices, it will be good to use legal grounds for punishment of family who practice open field defecation…’ (Woman FGD informant, age 56)

### Normative, ability, and self-regulation factors for improved F&E practices

**Fig. 6:**
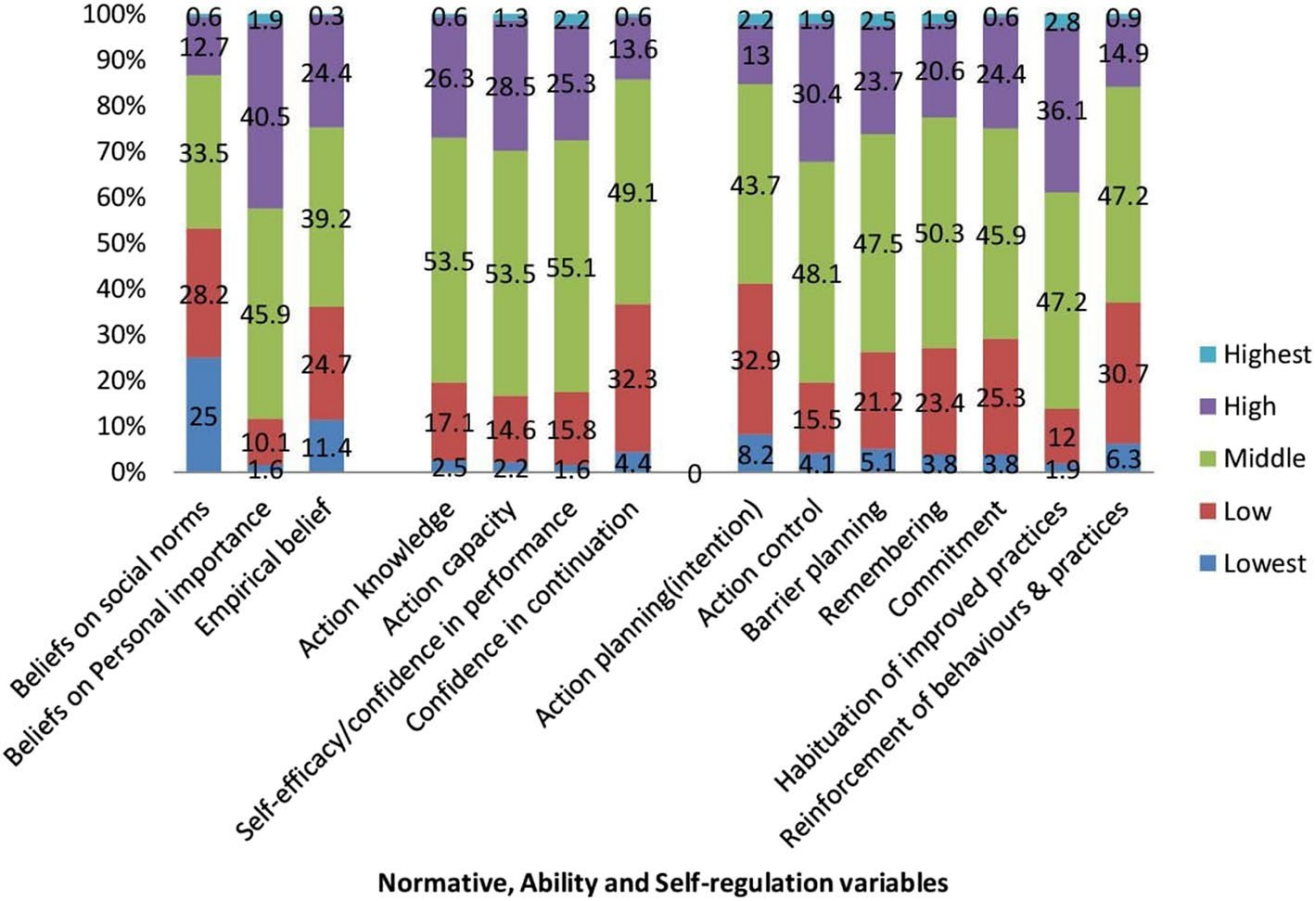
Normative, ability, and self-regulation factors among the study participants (*n* = 632) for improved F&E trachoma control practices in Southern Ethiopia

Many participants reported ‘medium response’ while asked for their normative, ability, and self-regulation factors. The ‘beliefs on social norms (33.5%)’, ‘beliefs on personal importance (45.9%)’, and empirical beliefs (39.2%) were among the reported ‘medium responses’ for normative beliefs. The ‘action knowledge (53.5%)’, the ‘action capacity (53.5%)’, ‘confidence in performance (55.1%)’, and a ‘confidence in continuation (49.1%)’ were ‘medium response’ reported by the majority for ability factors where confidence in one’s ability to practice behavior. The ‘action planning/intension (specification of when, where, and how to perform improved F&E practices) (43.5%)’, the ‘Self-monitoring and effort to continuously evaluate ongoing improved F&E-related practices (48.1%)’, the ‘Extent to which one attempts to plan to overcome barriers which would impede the adoption/execution of improved F&E practices (47.5%)’, the ‘perceived ease of remembering to practice the improved F&E behavior in key situations) (50.3%)’, the ‘commitment/obligation one feels to practice the improved F&E-related behaviors (45.9%)’, the ‘habituation of improved practices (47.2%)’, and re-enforcement of behaviors and practices (47.2%) were reported ‘medium responses’ by the majority of the participant while asked for self-regulation factors or attempts to plan and self -monitor behavior and to manage conflicting goals and distracting cues (Fig. 6).

### Clean body-beyond faces and clean environment – beyond latrines for improved practices

#### Quantitative arm

Of the study respondents (*n* = 632), only 50 (7.9%) households had improved practices for F&E trachoma control. More specifically, about 88 (13.9%) of the child washed their face and were visibly clean on the day of the interview. About 152 (24.1%) participants in the household reported as their child washed their face on the day of the interview. As parents reported, among those children who washed at any frequency, about 8.2% dried their face using a dried towel or cloth. By observation (interviewer) of face, the majority of the face was either ‘visibly clean’ or ‘somewhat clean’ as any one of the children in the household seen, 540(85.4%). Among those who reported daily face washing 302 (47.8%), and around 180 (59.6%) reported as it was incorporated into other relevant daily activities such as worshiping and school. About 96 (15.2%) of the households had a clean environment (composite indices). More than six out of ten households had no either human feces properly disposed of (62.7%) or a well-functioning latrine (64.9%). The living environment free of feces was 29.1% of households surveyed (Table 4).

**Table 4:** Clean body-beyond faces and clean environment – beyond latrines among the study participants (*n* = 632) for improved F&E trachoma control practices in Southern Ethiopia

**Variables**	**Number**	**Percent**
**Face washing experiences**		
The child washed face today		
Yes	152	24.1
No	480	75.9
How clean the face is by observation		
Visibly Clean	138	21.8
Somewhat clean (visibly clean, but fly on face)	402	63.6
Not clean	92	14.6
How frequently the child face washed in a week		
Once-two times a day	302	47.8
Once every other day	316	50.0
Once a week	14	2.2
What makes it easier to do face washing to your child daily? (*n* = 302)		
Water is easily available	60	19.9
It has been incorporated into other relevant daily activities	180	59.6
Others	62	20.5
What makes it harder to do face washing to your child daily? (*n* = 330)		
Water is not easily available	280	84.8
It has not been incorporated into relevant daily activities in the community	50	15.2
**Face-drying (shaking or with a cloth)**		
After face wash, do the child dry or wipe face		
Yes	52	8.2
No	580	91.8
Is the drying towel or cloth or material clean? (Observe) (*n* = 52)		
Visibly Clean	28	53.8
Somewhat clean	22	42.4
Not clean	2	3.8
Child Hand-washed with soap or other today?		
Yes	168	26.6
No	464	73.4
**Environmental cleaning experiences**		
Human feces is properly disposed		
Yes	236	37.3
No	396	62.7
Well-functioning latrine		
Yes	222	35.1
No	410	64.9
Keeping latrines clean, functional and accessible? (by observation) (*n* = 222)		
Yes	158	71.2
No	64	28.8
Keep the living environment free of feces (animal and human)? (by observation)		
Yes	184	29.1
No	448	70.9

Others = daily routine/habit, to be healthy and self-protection

#### Qualitative arm

The two KIIs reported lifestyles of inhabitants (congested, living with the animal) are the challenges to practicing improved sanitation and hygiene. Cultural acceptance of open-field defecation in the community and the cost of construction materials are some major challenges that favor unimproved practices. The two KIIs reported as:

‘Congested living situation in the household, low socio-economic status, being agrarian and pastoral farming are problems for improved practices. Open defecation is widely practiced. The problem of open defecation is due to the scarcity of money among household members to pay for standard latrine construction. Dwellers fear construction materials could be costly to construct a latrine. Traditionally accepted open defecation practices are optional and not encouraging the family to construct their latrine as well. The majority had a minimum interest in the construction of the latrine. The importance of latrine is not clearly understood though the information has been offered so far by HEWs and sanitation technicians…’ (42 years, male, district CDC coordinator)

‘Most inhabitants had no own latrine. The major problem of not using a latrine is due to cultural norms which are associated with past practices by the elders in most of the kebele, especially Jarso kebele. Most of the inhabitants in Jarso village defecate in the open field. No one denies open defecation in the area…’ (30-year-old, male, zonal NTD coordinator)

### Association of improved F&E trachoma control practices and predictor variables

#### Quantitative arm

A binary multivariable logistic regression output showed the association between improved F&E practices and the predictor variables, such as wealth index; awareness of trachoma transmission status; awareness of trachoma-related health risks; a stance toward a certain behavior; normative factors of social pressure towards a behavior; and ability factors of one’s ability to practice behavior. The occupation of the participant, understanding, and awareness of related health risks, Confidence in performance, and personal attempts to plan/self-monitor a behavior were associated only in the un-adjusted model.

The odds of having improved F&E trachoma control practices were 92 and 87% less likely in the ‘Low’ and ‘high’ wealth index participants than in those ‘highest’ (AOR = 0.08, 95%CI: 0.03, 0.28) and (AOR = 0.13, 95% CI: 0.05, 0.34), respectively.

The participant who was knowledgeable about trachoma transmission from person to person was 3.73 times more likely to have improved F&E trachoma control practices than their counterparts (AOR = 3.72, 95% CI: 1.6, 8.6).

#### Qualitative arm

Most informants stressed being aware of improved F&E practices, is not a problem. Everybody knows why he/she should wash her face and keep the environment clean. Inhabitants’ lack of essential knowledge and behavioral change in improved practices should be blamed.

‘I don’t believe the community has insufficient awareness about the benefit of face washing and environment cleanliness. The problem is behavioral change practices and disappointment due to water inadequacy. The children forcibly normalized not to wash their faces due to this reason. Having an inadequate understanding of the importance of face washing and a clean feces-free environment is also another thing seen among the inhabitants. (Male 30-year-old key informant)’

#### Quantitative arm

The participant who had a positive stance toward preventive behavior was four times more likely to practice improved F&E trachoma control practices than their counterparts (AOR = 3.94, 95%CI:1.59, 9.8). Odds of having improved practices among the participants who reported positive normative preventive behavior were 67% lower than those of negatives (AOR = 0.33, 95% CI: 0.13, 0.83). Good perceived ability among the participants was associated positively with improved practices than the poor ability to perform F&E (AOR = 2.31, 95%CI: 1.09, 4.88) (Table 5).

**Table 5:** Adjusted and unadjusted logistic regression model to show the association of improved F&E trachoma control practices in Southern Ethiopia

Variables	Improved F&E practices (*n* = 632)		COR (95%CI)	AOR (95%CI)	*P*-value
	No, n (%)	Yes, n (%)			
Occupation					
Farmer	422 (66.8)	30 (4.7)	1.00	1.00	
Merchant	54 (8.5)	8 (1.3)	2.1 (0.9, 4.8)^a^	1.9 (0.67,5.2)	0.23
Government employed	38 (6.0)	4 (0.6)	1.5 (0.49, 4.4)	0.9 (0.21, 3.5)	0.84
Others	68 (10.8)	8 (1.3)	1.6 (0.7, 3.76)^a^	1.1 (0.4, 2.8)	0.85
Wealth index					
Lowest	86 (13.6)	18 (2.8)	0.6 (0.29, 1.26)	0.58 (0.25, 1.37)	0.22
Low	122 (19.3)	4 (0.6)	0.09 (0.03, 0.29)^a^	**0.08 (0.03, 0.28)**^**b**^	*P* < 0.001
Middle	150 (23.7)	0	0.000	0.000	0.995
High	172 (27.2)	10 (1.6)	0.17 (0.07, 0.39)^a^	**0.13 (0.05, 0.34)**^**b**^	*P* < 0.001
Highest	52 (8.2)	18 (2.8)	1.00	1.00	
Knowledge of transmission from person to person					
Not knowledgeable	402 (63.6)	26 (4.1)	1.00	1.00	
Knowledgeable	180 (28.5)	24 (3.8)	2.1 (1.15, 3.7)^a^	**3.72 (1.6, 8.6)**^**b**^	0.002
Understanding and awareness of related health risks					
No	238 (37.7)	28 (4.4)	1.00	1.00	
Yes	344 (54.4)	22 (3.5)	0.54 (0.3, 0.97)^a^	0.68 (0.3, 1.6)	0.38
Stance toward preventive behavior (attitude)					
Negative stance	210 (33.2)	8 (1.3)	1.00	1.00	
Positive stance	372 (58.9)	42 (6.6)	2.96 (1.37, 6.4)^a^	**3.94 (1.59, 9.8)**^**b**^	0.003
Perceived social pressure towards a behavior; other’s actions and opinions regarding a behavior					
Negative	278 (44)	38 (6)	1.00	1.00	
Positive	304 (48.1)	12 (1.9)	0.3 (0.15, 0.56)^a^	**0.33 (0.13, 0.83)**^**b**^	0.018
Perceived action capacity/ability to perform					
Poor	418 (66.1)	26 (4.1)	1.00	1.00	
Good	164 (25.9)	24 (3.8)	2.3 (1.3, 4.2)^a^	**2.31 (1.09, 4.88)**^**b**^	0.029
Confidence in performance					
Poor	428 67.7)	30 (4.7)	1.00	1.00	
Good	154 (24.4)	20 (3.2)	1.8 (1.0, 3.4)^a^	1.84 (0.8, 4.21)	0.15
Self-regulation-attempts to plan and self -monitor a behavior					
Poor	268 (42.4)	28 (4.4)	1.00	1.00	
Good	314 (49.7)	22 (3.5)	0.67 (0.37, 1.2)^a^	0.82 (0.33, 2)	0.66

^a^Significant only in binary LR

^b^Significant in binary and multivariate LR; and others include daily laborer, housewife, student, NGOs employed

#### Qualitative arm

Interest in face washing and environmental cleanliness is frequently described by the informants and discussants in their interview reports. Though caretakers perceive that the child knows to wash his/her face, he/she is not interested in practicing. The caretaker doesn’t care about that. One informant said:

‘Attitude is another problem in the family. Face-washing skill is not a problem. Every family should be able to wash their child. I believe that the elderly baby also has the skill but had no interest to practice face washing and toilet use in defecation.’ (40 years old male key informant)’

And, most FGD discussants reported, that caretaker recklessness was out shinning to care for their child’s hygiene and sanitation. One discussant reported:

‘Unwashed (Dirty face) of the child is not normally accepted by the community. The problem is no one cares about a child with a dirty face to clean him/her. In my opinion, the village people consider it the child’s responsibility to clean him/her in the community.’ (discussant1)

### Barriers to improved F&E practices

#### Contextual barriers

Even though clean water shortage was mentioned repeatedly by informants in the village, being arid/dry land and the deeply rooted underground water table is a challenge to get water access easily. One FGD informant reported:

‘ohhh…. The shortage of clean water in the village is a challenge. We use seasonal river water. The river exists during the rainy season and disappears during the dry season. Our exposure to trachoma is high because we and our children are not washing faces frequently with water and soap. Trachoma is more prevalent in the village. We see many people with trachoma-related eye problems in our neighborhood. It takes a long time to fetch water. Our children do not wait until the water is brought from the river to go to school or farm with cattle. So, usually, they depart without washing their face. They eat their breakfast without washing their face. (70-year-old elder informant)’

Two key informants working in the district as NTD and ORBIS international Ethiopia focal person added hydrogeological barriers to getting water in the village. They mentioned:

“Being arid/dry land and the deeply rooted underground water table is a challenge to get water access easily. Shortage of rivers is another to be considered in the district and zone as a whole. The community cannot get water even digging into the land very deeper. If no water, it is much more difficult to think even about clean practices. Water is the center of gravity in all clean hygienic practices”

#### Psychosocial barriers

Parents’ negligence of not caring, clean water access problem, inadequate motivational factors, prioritization, and living together with cattle in one home makes the improved practice poor in the village. One farmer informant among the discussants reported:

‘For face washing, we use only water but not soap. Personal hygiene is not something to be given priority. We have many other issues that need priority. If we can get water we wash our face otherwise we don’t. Many times our children go farming without washing their faces since there is no water. The existing minimum water supply is even not used properly. Since we are living together with cattle, it is difficult to create a clean environment and face. (52-year-old farmer discussant)’

Regarding proper Latrine usage for a clean environment, informants reported as they are more responsible for unimproved practices than local authorities. They declared as they have got frequent practical information from Health extension workers, authorities, NGOs, and village sanitation technicians. The word ‘norm’ and ‘behavioral change’ were highly repeated terms raised as the major challenge to better hygienic practices. One informant reported:

‘We do not say local authorities are responsible for our unimproved hygiene and proper latrine use. They did a positive effort. The problem is our community. They do not obey. They defecate on the field, farmland, river, and street. Some family has functional toilet but they often do not use them. They prefer field defecation to the toilet. People / Members of our community like to defecate on the field. The reason is not known. It is a habit for a long ago. Long-term and sustainable behavioral change practices may solve the problem. The existing water shortage is even not used properly for sanitation. In most villages, it is seen norm to defecate in the toilet for elder children and adults. So, they go to the field to defecate. And, we cannot afford the cost of toilet construction materials at the family level. The economy is another problem. The other issue is if the toilet is constructed and is open with the minimum number of wood Slabs, it could be dangerous to our children, cattle, ships, goat, and chickens in that they may fall into it. Having no recognition and reward policy for few of inhabitants who constructed latrines would have been discouraging others(not have latrines) and children to bring behavioral change for improved overall practices of F&E.’ (59-year-old informant)

#### Technology barriers in latrine construction

Reflection on the cost of construction material is used most frequently by informants. Inhabitants consider themselves incapable to buy and pay for a builder of this highly escalated material to build a good latrine. A latrine built with wood and dust is short-lived. One informant reported:

‘Some construction material cost is the barrier to making the good latrine construction that they want. Such as cement cost, metal works, and others. Locally available latrine construction materials are used to build only short-lived latrines. That disappoints them to construct.’ (37-year-old informant)

### Facilitators of F&E trachoma control practices

The most frequently described encouraging factors for improved practices are the existence of community latrines, health extension workers’ continued effort to disseminate health information on the benefit of being clean, and drugs given for trachoma mass drug administration for prevention in children and adults. The effort by ORBIS is not negligible. One informant said:

‘Improved practices may be facilitated by…offering some construction materials at low costs, such as Slab for latrine construction is encouraging, but most people still do not buy and use it. Orbis international Gamo Gofa branch supported some facilitation, such as building some communal latrines in the village and supplying Azithromycin Trachoma drug with awareness creation information to the children. UNICEF Wash Support is another strengthening facilitator which is seen positively by the inhabitants in that they give training to some village inhabitants to construct their latrines and be a model in the village. They also recruited some motivating and informing technicians in the village to facilitate, support, encourage, coach, and consult the community for latrine construction and hygienic practices. Reduction of the prevalence of active trachoma is an encouraging facilitator though it was not achieved in the manner planned as the target by W.H.O. Existence of some model latrines graduation ceremonies at the kebele could have been encouraging practices to others to build their latrines. The existence of eye health workers in the kebele could be encouraging for facial hygienic practices.’ (42-year-old district communicable diseases coordination key informant)

## Discussion

This study tried to assess the status of the improved face and environmental hygienic practices in one of the trachoma endemic areas of Southern Ethiopia. It was reasoned that trachoma eliminated in many areas of the U.S.A and Western Europe before the emergence of antibiotics was to the establishment of better hygienic and sanitary improvement practices [22]. The observed improved practices for F & E in this agro-pastoral study area (7.9%) were much lower than the SAFE strategy F&E improvement plan (80%) for successful elimination practices of trachoma [23]. When specifically observing, the proportion was much lower than the prevalence seen by ORBIS international findings of the same but broader regions of Southern states, such as access to the latrine (95 vs 35.1%) and facial cleanliness (69 vs 21.8%) [9]. Limited access to clean water (such as 15.2% access to protected water) and longer time to fetch (> 1/2 h, 78.2%), minimal awareness of trachoma prevention strategies of F&E in SAFE (17.1% aware), and attitude (34.5% with negative attitude) could be blamed for huge un-improved practices observed in the study locality. Some of these are in-line and others are opposed (awareness) with qualitative findings in that ‘Most discussants said awareness is not the problem in the village. The problem is behavioral change in communication and attitude in children and parents for improved practices. The scarcity of clean water and a longer time to fetch river water is another challenge mentioned.

According to the bulletin of W.H.O., using mean household count wealth indices, community-level poor sanitation was related to the lowest wealth index of the same level in Ethiopia, Amhara [24]. This was fitting with the current study. Kenyan recent DHS finding also supported the current finding [25]. Challenges to improved hygienic practices in poor countries, like Ethiopia, are related to the shortage of clean and adequate water supply, cultural and behavioral perspectives of hygiene, low functioning of hygiene workers, problems of information dissemination on its infrastructure and behavioral change work, problems of gender inclusion (such as, excluding women from participation), problems of inhabitants participation in the planning phase, lack of political commitment (leadership role), and poverty [26, 27].

As seen in this study, being knowledgeable about trachoma transmission from person to the person had a positive association with improved F&E trachoma control practices. The existing knowledge could lead to protecting behavior in that human being naturally seeks safety with healthy living. This was also supported by a study done in rural Tigray, a regional state in Northern Ethiopia, where those knowledgeable were three times more likely to practice F&E-improved practices [28]. It is also consistent with the guideline of the International Coalition for Trachoma Control that long-term education or awareness programs are one strategy to offer knowledge on the prevention and control of trachoma [15]. This was supported by each other in the qualitative arm in that inhabitants’ shortage of basic knowledge, attitude, and behavioral change for improved practices were reported by the key informants. They are aware but do not practice or intend to practice.

Behavior change communication to bring a positive attitude brings good achievement in clinical settings and public health in the prevention of both communicable and non-communicable diseases [29]. The fact was agreeing with the findings that having a positive stance towards preventive behavior had a positive association with improved F&E trachoma control practices. This is because the predecessor is influencing the successor, as is also mentioned in the chains of the theory of triadic influences [17].

According to a finding from a study conducted in central Ethiopia, some social trends favored the practices of improved facial and environmental cleanness [20]. But, in this study, having positive “perceived social pressure towards a behavior; other’s actions and opinions regarding a behavior” was negatively associated with improved practices. This opposite relationship could be related to the community belief or trust in health professionals’ information and pressure rather than peers or societies in the study locality. Belief in health professionals’ information and pressure for a healthy lifestyle in disease prevention and control was high as witnessed by one study in southern Ethiopia [30]. This was also true by key informants and FGD reporters in that facilitators for improved practices of sanitation and hygiene in the district are governmental health office workers, NGO workers (UNICEF and ORBIS), and Health Extension Workers. No informant reported any perceived social pressure as facilitators to improved practices. Suppose an unwashed face is not accepted in the community but no one cares about it, meaning it has no effect as social pressure on the children and parents, as said by discussants.

In this study, good perceived ability among the participants was associated positively with improved practices. This was in line with CDCs and other trachoma control and prevention behavior recommendations [31, 32]. This shows that one’s ability or skill makes it protects oneself from infectious diseases, such as trachoma.

### Strengths of the study

Using triangulated design might be a benefit than making quantitative alone in that findings in both techniques helped to observe either contradicting or in-line to each other and some additional barriers and facilitators tried to assess by the qualitative arm. Observational data collection techniques used to collect facial and environmental cleanliness and clean water sources data makes it stronger than those collected by interview reports alone.

### Limitations of the study

Participants hiding the real scenario of malpractices to the educated interviewer to some interview questions could affect the result to some extent. Using observational data minimized this bias. The interview process could have been disturbed to some extent because the scared interviewee of Covid-19 transmission through infection prevention mechanisms had been performed such as using masks, alcohol hand rub, and keeping social distancing between interviewer and respondent.

## Conclusions and recommendations

F&E improved trachoma control practices in the rural locality of the study area were much lowest than the regional plan for successful elimination practices of trachoma by the SAFE strategy. The scarcity of clean water was the major issue observed in the locality. Four out of five caretakers had awareness of trachoma eye disease. About two-thirds of participants believed that trachoma is transmitted by dirty face and/or flies and the prevention was by surgery and/or drugs (antibiotics). Participants’ cost belief towards improved practices for F&E trachoma control was the lowest. Satisfying response to normative, ability, and self-regulation factors for improved practices was observed by nearly half of the participants. Wealth index; awareness of trachoma transmission status; awareness of trachoma-related health risks; a participant’s stance on some particular behavior; normative factors of perceived social pressure towards a behavior; and ability factors of one’s ability to practice improved behavior were potent predictors of improved F&E practices for trachoma prevention and control. Community-based mass sanitation and face washing campaign should be done in the community at least weekly through the collaboration of health care workers. Model households should be selected so that behavioral change intervention should be done house to house for environmental and facial improvement practices. Parent attitude-changing knowledge and practices should be encouraged. The scarcity of clean water supply should be given due attention to the improvement of water supply shortages in the community, increasing groundwater supply, so it will be easy to facilitate hygienic practices.

## Data Availability

All data generated or analyzed during this study are included in this published article.

## Abbreviations

F&E: Face hygiene and Environmental cleaning
FGD: Focus Group Discussion
HCW: Health Care Workers
HF: Health Facility
KII: Key Informant Interview
NTDs: Neglected Tropical Diseases
SNNPR: Southern Regional State of Ethiopia
TI: Trachomatous Inflammation – Intense
TF: Trachomatous inflammation – Follicular
TTIs: Theory of Triadic Influences
